# The Cortical Contributions to Turning Performance Through Muscle Synergies in Parkinson’s Disease: A Mediation Study

**DOI:** 10.3390/bioengineering13040453

**Published:** 2026-04-13

**Authors:** Mirabel Ewura Esi Acquah, Zengguang Wang, Wei Chen, Dongyun Gu

**Affiliations:** 1 School of Biomedical Engineering, Shanghai Jiao Tong University, No. 1954 Huashan Road, Xuhui District, Shanghai 200030, China; miry_a@sjtu.edu.cn (M.E.E.A.);; 2 Department of Orthopedics, Shanghai Ninth People’s Hospital, No. 833 Zhizaoju Road, Huangpu District, Shanghai 200011, China; 3 Department of Neurology, Shanghai Ninth People’s Hospital, No. 833 Zhizaoju Road, Huangpu District, Shanghai 200011, China

**Keywords:** Parkinson’s disease, functional connectivity, muscle synergy, turning, motor control, mediation analysis

## Abstract

Turning impairment is a major contributor to falls in Parkinson’s disease (PD), yet the mechanisms linking cortical dysfunction to altered motor behavior remain unclear. In particular, it is unknown whether disrupted cortical communication impairs turning by altering muscle coordination. This study investigates a novel mechanistic pathway: whether muscle synergy complexity mediates the relationship between cortical network connectivity and turning performance in PD. Specifically, electroencephalography (EEG) and electromyography (EMG) were recorded from 12 individuals with PD and 12 age-matched healthy controls during a 180° turning task. Directed cortical connectivity, muscle synergy complexity, and spatiotemporal turning performance were quantified. Mediation analysis was used to determine whether cortical influences on behavior operate indirectly through neuromuscular coordination. Compared to controls, individuals with PD performed slower turns with shorter stride lengths and reduced synergy complexity (*p* < 0.05), alongside altered frontal cortical connectivity (*p* < 0.05). Across participants, higher synergy complexity was associated with faster, longer strides (*p* < 0.04). Cortical connectivity strength strongly predicted synergy complexity (R^2^ = 0.66, *p* < 0.001) and exerted a significant indirect effect on turning performance (β = 0.312; 95% CI [0.072, 0.605]; *p* = 0.008). In PD, reliance on this indirect pathway increased with disease severity and poorer turning ability (r > 0.57, *p* < 0.03). This work establishes how muscle synergy complexity significantly mediates the relationship between cortical connectivity and turning performance in PD. Our findings provide evidence of a cortical–neuromuscular–behavioral pathway underlying turning deficits, highlighting coordination as a key target for neurorehabilitation.

## 1. Introduction

Parkinson’s disease (PD) is a common neurodegenerative disorder characterized by severe motor impairments, including freezing of gait, tremors, bradykinesia, rigidity, and postural instability [1,2]. Among these, turning deficits are particularly disabling and represent a leading cause of falls, since turning places substantially greater demands on cognitive processing and neuromuscular coordination compared to straight walking [3,4,5]. Despite their clinical importance, the mechanisms underlying impaired turning in PD remain poorly understood.

For every movement task, effective motor control relies on coordinated interactions between cortical networks and the neuromuscular system. Unfortunately, PD is characterized by abnormalities in both cortical dynamics [6,7,8,9] and muscular activation [10,11,12], and these impairments have been linked to reduced gait performance [13,14,15]. Additionally, individuals with PD appear to recruit alternative cortical pathways, suggesting compensatory reorganization [14,16], alongside increased EEG complexity in frontotemporal and sensorimotor regions during movement [17,18,19]. Nonetheless, an essential unresolved issue is whether these cortical disruptions lead to motor impairments through altered neuromuscular coordination or represent parallel or independent deficits.

Recent work has advanced our understanding of brain-muscle interactions during walking in PD. For example, Roeder et al. demonstrated tighter brain–muscle coordination patterns with aging and PD, while Pellegrini et al. reported reduced information transfer from the supplementary motor area during hypokinetic gait [20,21]. Although these studies establish associations between cortical activity and motor performance, they do not test whether neuromuscular coordination constitutes the mechanistic pathway linking brain dysfunction to impaired movement. Moreover, this line of research has been largely confined to straight walking, leaving the more complex and fall-prone task of turning unexamined.

Muscle synergies provide a means for understanding how the nervous system coordinates groups of muscles to produce efficient movement [22]. The number and structure of these synergies indicate the complexity and adaptability of neuromuscular control [23,24,25]. In PD, gait is consistently characterized by fewer and more rigid muscle synergies, indicating reduced coordination complexity and limited adaptability [26,27,28]. Previous studies further suggest that these synergies are shaped by common cortical inputs, particularly from motor cortical regions [29,30]. Together, these findings support the view that impaired motor performance in PD may arise from simplified neuromuscular coordination driven by altered cortical activity. However, existing muscle synergy studies in PD have focused on straight-line walking and postural tasks [31], with turning remaining entirely uncharacterized. Specifically, whether the proposed cortical–neuromuscular link is preserved or disrupted during turning in PD remains unknown.

To address this gap, we introduce a multimodal mediation framework that explicitly tests the cortical–neuromuscular–behavioral pathway underlying turning performance. By integrating measures of directed cortical connectivity, muscle synergy complexity, and spatiotemporal turning metrics, this approach moves beyond pairwise associations to directly test a mechanistic pathway in PD. Furthermore, by quantifying how reliance on this indirect pathway varies with disease severity, the study provides insight into individual differences in motor control strategies and their progression. We hypothesize that the muscle synergy complexity mediates the relationship between cortical connectivity and turning performance. In PD, impaired turning may result from reduced coordination complexity due to changes in cortical network interactions. Understanding this pathway is essential for developing neurorehabilitation approaches that aim to restore coordination rather than simply compensating for its loss.

## 2. Methods

### 2.1. Participant Characteristics

This study was conducted in the Human Movement Laboratory at Shanghai Jiao Tong University, China. Twelve individuals with PD and twelve healthy age-matched adults were included in this study. A minimum of 12 participants per group was chosen in line with established conventions for pilot multimodal EEG–EMG studies [32]. All PD participants were referred to the experiment from the Shanghai Ninth People’s Hospital after undergoing the Unified Parkinson’s Disease Rating Scale (UPDRS), Hoehn and Yahr (H&Y) staging, Mini-Mental State Examination (MMSE), and Montreal Cognitive Assessment (MoCA) to assess overall clinical status and disease severity. Participants were enrolled according to the following inclusion criteria: (1) age between 55 and 75 years, (2) right-handed according to the Edinburgh Handedness Inventory, (3) can walk and turn independently, (4) no history of other cognitive, psychiatric, or neurological illnesses apart from PD, and (5) no orthopedic conditions or significant disease affecting gait or balance apart from PD (Table 1). All participants in the PD group were identified as freezers (i.e., they experienced freezing of gait when walking). This patient group was assessed after taking their dopaminergic medications, with an average daily levodopa-equivalent dose of 350 ± 254 mg/d.

The experimental procedures were explained to all participants, who signed informed consent forms before the experiment. Before this study was conducted, the Ethics Review Committee for Science and Technology Research Involving People at Shanghai Jiao Tong University (No. E20200351) approved it. All methods were performed in accordance with the relevant guidelines and regulations.

### 2.2. Experimental Setup

**Figure 1:**
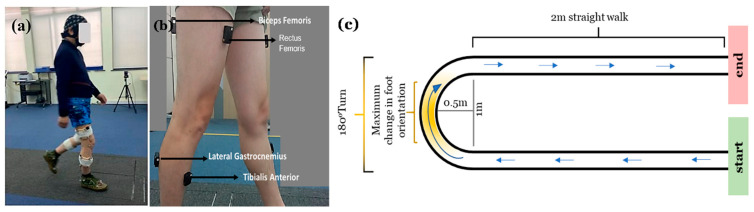
(**a**) shows a participant performing the experiment trial; (**b**) shows the EMG placements of the muscles measured. Eight lower limb muscles were measured (4 on each limb). (**c**) shows the walking path with a 180° turn. The blue arrows in the task represent the direction of the participant’s walk. This setup captures simultaneous multimodal neural, muscular, and kinematic data during a standardized, ecologically valid turning task.

From the starting line, participants walked 2 m to a demarcated point, performed a 180° right turn (radius = 0.5 m), and returned to the starting line (Figure 1c). Using a fixed turning direction minimized variability from geometry and footedness. Each participant completed ten self-paced trials without assistance.

Spatiotemporal kinematic data were captured using an eight-camera motion capture system (Vicon Nexus 1.8.5 System, Vicon Metrics Ltd., Oxford, UK; Sampling rate: 100 Hz). Six reflective markers were bilaterally placed on the toe, malleoli, and heel to determine gait phases. Simultaneously, eight surface EMG sensors recorded bilateral activity from the tibialis anterior (TA), lateral gastrocnemius (GL), rectus femoris (RF), and biceps femoris (BF), covering both distal and proximal muscle groups involved in propulsion and stability. This bilateral configuration was deliberately chosen to capture the biomechanical asymmetry inherent to turning. Electrode placement followed SENIAM guidelines (http://www.seniam.org/).

### 2.3. Data Analysis

Since this study involved a multimodal dataset combining neural, muscular, and behavioral measures, we performed several analyses for each modality before investigating the relationships among cortical connectivity, muscle coordination, and gait performance.

#### 2.3.1. Turning Period Definition

**Figure 2:**
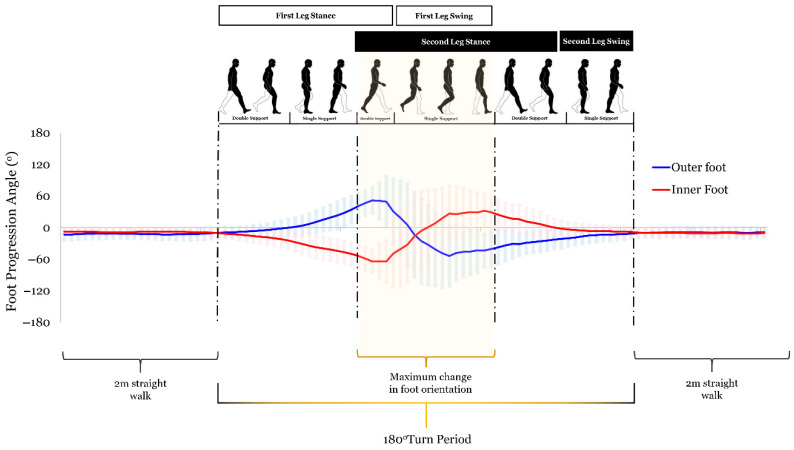
The change in foot progression of healthy participants as they walk through the designated pathway. The turning period, with its gait phases, was extended to capture the maximum change in foot orientation. The outer foot (left) trajectory is shown in blue, and the inner foot trajectory (right) is shown in red. The peak section of the turn is highlighted in yellow. This two-stride window ensures both the inner and outer limbs’ distinct mechanical contributions are fully represented in the analysis.

Analysis focused on the 180° turning phase, a task requiring higher bilateral coordination and flexibility than straight walking. The turning period was defined as the two consecutive strides surrounding the point of maximum change in foot orientation, as derived from the foot progression angle line [33]. This period, spanning from the initiating limb’s heel strike to the contralateral limb’s second heel strike, captured both limbs’ contributions across the full turn (Figure 2).

Since the left and right limbs follow distinct trajectories with different biomechanical functions, they were analyzed separately as the outer (left) and inner (right) legs. Turning performance was quantified using outer and inner stride lengths and overall turning speed.

#### 2.3.2. Muscle Synergy Analysis

The collected EMG signals were band-pass filtered (20–500 Hz), demeaned, rectified, and smoothed for each subject trial [34]. These clean signals were then segmented according to the defined turn periods, enveloped, and time-normalized to fit a 100-point base. Before synergy extraction, EMG signals were standardized to unit variance. Outer and inner leg muscles were treated as functionally distinct due to their differing biomechanical roles during the segmented period, yielding 8 muscle envelopes in total, with 100 sample points (8 × 100) per trial. This approach differs from conventional synergy analyses of straight walking, which typically pool ipsilateral muscles or treat gait as symmetric. By retaining the full bilateral muscle set, the synergy decomposition captures coordination strategies that span both limbs throughout the turning task, in which each limb performs a fundamentally different mechanical function. This design choice is supported by prior work demonstrating that turning engages distinct bilateral muscle coordination patterns not observed during straight walking [24].

Muscle synergy was derived using the non-negative matrix factorization method (NNMF) to decompose the preprocessed EMG signals into spatial and temporal synergy structures [24,35]. Analyses were performed at the individual level to capture subject-specific coordination patterns. For each subject, all preprocessed EMG trials were concatenated into a single continuous matrix for decomposition. NNMF decomposition was repeated 30 times per subject with independent random seeds, and the solution yielding the lowest reconstruction error was retained to ensure a stable, reproducible decomposition.

To quantify the neuromuscular complexity of each subject’s muscle synergies, we calculated standard complexity metrics from previous studies [25,36], including: Variance Accounted For with only one synergy (VAF1) and the minimal synergy number (MSN, i.e., the smallest number of synergies required to explain at least 90% of the total variance). To confirm the stability of MSN values, synergy solutions were extracted across a range of synergy numbers (2–8) per subject.

#### 2.3.3. Cortical Functional Connectivity

EEG preprocessing was performed in EEGLAB [37]. Data were band-pass filtered (0.1–30 Hz), line noise removed (50 Hz notch), and artifacts corrected using Artifact Subspace Reconstruction (ASR). Signals were then re-referenced to the average reference and segmented by turning period.

Directed functional connectivity was estimated using Partial Directed Coherence (PDC) to quantify interactions between brain regions at different frequencies [38,39], yielding 3D matrices (channels × channels × frequency). The PDC connectivity matrices between 0–30Hz were averaged to produce subject-level 2D connectivity maps (64 × 64). The 0–30 Hz averaging range was selected to encompass the slow-oscillatory and beta-band activity previously linked to PD gait impairment [17,40,41]. The multivariate autoregressive model order for PDC estimation was determined per subject using the Akaike Information Criterion [39].

To assess the statistical significance of the observed connectivity matrices, a surrogate phase-randomized analysis was used to establish a 95th-percentile threshold, thereby removing spurious edges from the series [42,43]. From this mask, connectivity edges significantly correlated with synergy complexity and turning metrics (*p* < 0.05) were retained and weighted by correlation strength, yielding a final sparse, weighted matrix that emphasized functionally relevant connections.

The connectivity network strength was computed at global and regional levels. Global network strength was computed by averaging all non-zero entries in the PDC matrix, while regional strengths were defined for Frontal (Fp1, Fp2, AF3, AF4, F1, F2, F3, F4, F5, F6, F7, F8, Fz), Central (FC1, FC2, FC3, FC4, FC5, FC6, FCz, C1, C2, C3, C4, C5, C6, Cz, CP1, CP2, CP3, CP4, CP5, CP6) and Posterior (P1, P2, P3, P4, P5, P6, P7, P8, Pz, PO3, PO4, PO7, PO8, Oz, O1, O2) subnetworks and expressed as ratios of global network strength.

### 2.4. Statistical Analysis

#### 2.4.1. Group Comparisons

Group differences between PD participants and healthy controls were assessed for all features extracted at different levels in this study. The group differences in gait features (turning speed and stride length), synergy features (MSN and VAF1), and the network features (global network strength and regional strength ratios) were evaluated using independent two-sample *t*-tests.

#### 2.4.2. Multimodal Associations

Associations among neural, muscular, and gait metrics were explored using non-parametric and regression-based approaches. Kruskal–Wallis tests compared turning performance across synergy-complexity groups, with post hoc Dunn tests.

LASSO regression identified the EEG connectivity edges predictive of synergy complexity. Models were bootstrapped (1000 iterations) with ten-fold cross-validation to optimize the regularization parameter λ within each bootstrap iteration, and node stability was quantified by selection frequency. Model performance was additionally evaluated on held-out folds, yielding a mean out-of-sample R^2^ consistent with the reported in-sample fit, supporting the absence of gross overfitting. Connections surviving both bootstrapped stability selection (above the 75th percentile) and cross-validated regularization were retained, reducing the risk of spurious edge inclusion.

#### 2.4.3. Mediation Analysis

A LASSO-based parallel mediation model tested whether muscle synergy complexity mediated the relationship between EEG connectivity strength and gait performance, with MSN and VAF1 entered simultaneously as mediators [44,45,46]. Mediation analysis uses regression to divide the total effect of a predictor on an outcome into a direct effect and an indirect effect mediated by a third variable [47]. Including covariates in each regression helps isolate these effects from confounding, allowing us to estimate the extent to which the predictor’s influence is mediated. A parallel mediation model extends this by simultaneously assessing multiple mediators, enabling independent quantification of their contributions while controlling for one another. This parallel structure allowed the specific contribution of each complexity index to be estimated while controlling for the other, avoiding the interpretive limitations of single-mediator models. The model included: the regression of each mediator (MSN and VAF1) on connectivity strength (paths a1 and a2); the regression of gait performance on both mediators while controlling for connectivity (paths b1 and b2); the direct effect (c′) of connectivity on gait controlling for both mediators; and the specific indirect effects (a1 × b1 for MSN; a2 × b2 for VAF1). Indirect effects were estimated using 95% confidence intervals (5000 samples). A bootstrapped contrast test assessed whether the two indirect effects differed significantly.

To assess individual influence, a leave-one-out (LOO) approach was used to recalculate the mediation estimates, excluding one participant at a time [47,48]. Influence scores, defined as the proportional change in group-level mediation when each participant was omitted, were correlated with turning performance and clinical scales (UPDRS, H&Y, MMSE, MoCA, FOGQ) to examine whether reliance on the indirect pathway varied with disease severity. To select covariates for the mediation model, baseline characteristics including age, height, weight, MMSE, and MoCA were compared using *t*-tests. Variables with *p* < 0.20 were included to control for potential confounders and to estimate direct and total effects. MoCA differed significantly (*p* < 0.01) and was thus included as a covariate.

(1) $${Influence\;score}_{i}\;=\;\frac{Group\;mediation\;effect-\;{LOO\;mediation\;effect}_{i}}{Group\;mediation\;effect}$$

## 3. Results

### 3.1. Group Differences in Gait, Muscle Synergy, and Brain Connectivity

During the turning period, the PD group demonstrated significantly reduced spatiotemporal performance. Specifically, they exhibited slower turning speed (Cohen’s d = 0.97, *p* = 0.022) and shorter stride length (Cohen’s d = 0.89, *p* = 0.035) than the healthy group, particularly on the outer trajectory (*p* < 0.05) (Table 2).

**Figure 3:**
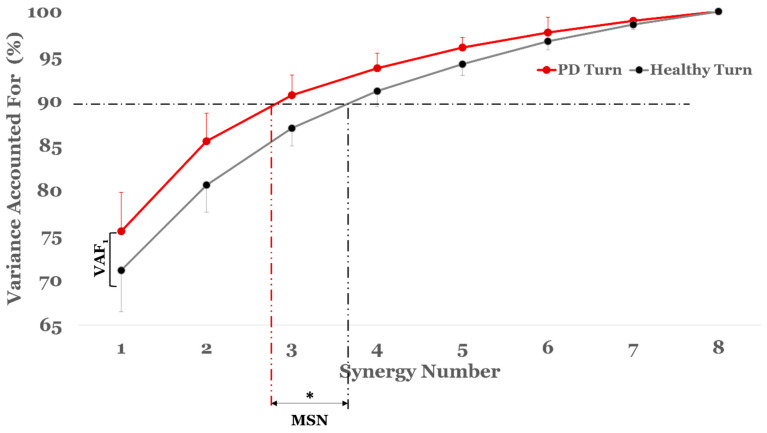
The synergy complexity is derived as the EMG variance explained by each synergy number for both healthy (black) and PD (red) participants. This figure also shows the differences between participant groups in synergy complexity features (VAF1—Variance Accounted For using only one synergy and MSN—Minimal Synergy Number). The dashed line (–·–) at 90% indicates the threshold used to identify MSN in both groups. Asterisk (*) represents statistical significance (i.e., *p* < 0.05). PD participants require significantly fewer synergies to explain the same amount of variance, indicating simplified and less adaptable neuromuscular coordination during turning.

The minimal synergy number (MSN) was significantly lower in the PD group compared to the healthy group during turning (Cohen’s d = 1.38, *p* = 0.002). While not statistically significant, the PD group also showed higher VAF1 values than healthy controls (Cohen’s d = −0.76, *p* = 0.057) (Figure 3; Table 2).

**Figure 4:**
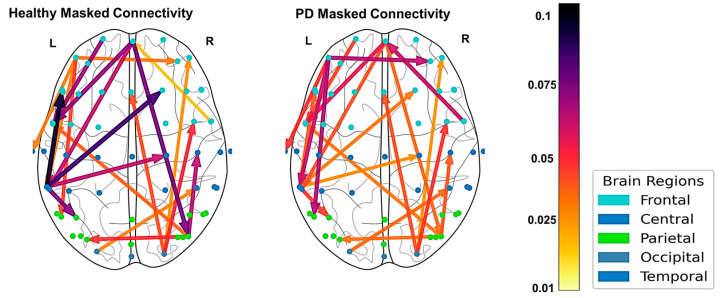
Group-level directed EEG connectivity during the turning period. Motor-relevant cortical connections that survive the surrogate- and correlation-based masking procedures are shown for healthy controls and individuals with PD. Edge thickness and color (arrows) reflect the strength of partial directed coherence (PDC). Node color (dots) denotes the cortical region of origin (see legend). L and R represent the left and right hemispheres. This shift toward frontal-dominant connectivity in PD is consistent with compensatory top-down motor control replacing the automatic subcortical pathways disrupted by the disease.

Although the global network strength of the selected network was not significantly different between groups (*p* = 0.21), regional strength analysis revealed the PD group exhibited significantly stronger frontal-to-frontal (Cohen’s d = −1.03, *p* = 0.017) and central-to-frontal (Cohen’s d = −1.05, *p* = 0.017) connectivity compared to healthy controls, but showed reduced connectivity from posterior to frontal regions (Cohen’s d = 0.88, *p* = 0.033) (Figure 4; Table 2).

### 3.2. Multimodal Associations

Having established significant differences between groups, we explored cross-modal associations across all participants.

#### 3.2.1. Turning Performance with Muscle Synergy

**Figure 5:**
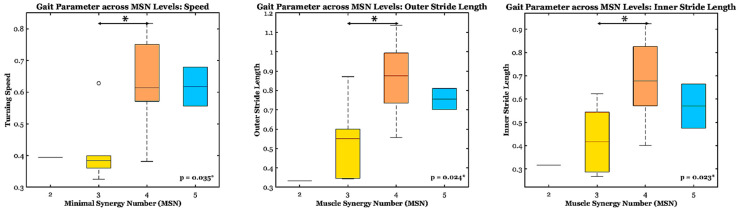
The gait parameters across Minimal Synergy Number (MSN) levels to estimate the relationship between MSN and speed, inner and outer stride length. Asterisk (*) shows significance (*p* < 0.05). Colors represent different levels of MSN (MSN 3—yellow, MSN 4—orange, MSN 5—blue). Higher synergy complexity predicts better turning performance across both groups, with the most pronounced effect on outer-leg stride length.

From the Kruskal–Wallis test, turning speed and stride length from the inner and outer legs differed significantly across MSN levels (*p* < 0.04). Post hoc analysis revealed that subjects with MSN of 3 and above exhibited significantly higher turning speed and longer stride length, especially in the outer leg, than those with MSN less than 3 (*p* < 0.03) (Figure 5). However, VAF1 did not appear to meaningfully capture this relationship (*p* > 0.4).

#### 3.2.2. Functional Connectivity with Muscle Synergy

Lasso regression with stability selection identified 15 functional EEG connections above the 75th percentile threshold. Together, these neural nodes collectively explained approximately 66% of the variance in MSN (R^2^ = 0.66, F = 8.21, *p* < 0.001). The strongest positive association was observed for PO8 → FC5 (coefficient = 0.81), while the strongest negative association was found for O1 → CP4 (coefficient = −1.07).

### 3.3. Mediation Analysis (Brain—Muscle Synergy—Turning Performance)

**Figure 6:**
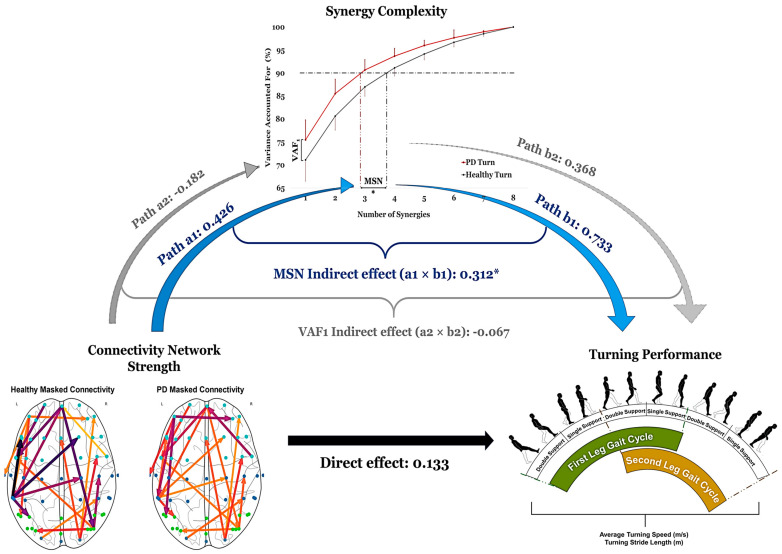
Parallel mediation model linking cortical connectivity to turning performance through muscle synergy complexity. Connectivity network strength was entered as the predictor, with the synergy complexity features, i.e., minimal synergy number (MSN) and Variance Accounted For with only one synergy (VAF1), simultaneously entered as mediators and turning performance as the outcome. The synergy complexity curve illustrates how each metric is derived: VAF1 represents the variance explained by a single synergy, and MSN denotes the minimum number of synergies required to explain 90% of the variance (vertical dashed lines). Blue arrows and bold blue labels indicate the significant indirect pathway through MSN (a1 × b1 = 0.312; 95% CI [0.072, 0.605]; *p* = 0.008). Grey arrows indicate the non-significant indirect pathway through VAF1 (a2 × b2 = −0.067; 95% CI [−0.273, 0.081]; *p* = 0.415). * *p* < 0.05. MSN, but not VAF1, is the active intermediary through which cortical connectivity influences turning speed, confirming the specificity of the MSN pathway.

Parallel mediation analysis, with MSN and VAF1 entered simultaneously, confirmed that cortical connectivity positively predicted MSN (path a1, β = 0.426) but showed a weaker relationship with VAF1 (path a2, β = −0.182). MSN was positively associated with turning speed while controlling for connectivity and VAF1 (path b1, β = 0.733), whereas VAF1 showed a weaker association (path b2, β = 0.368). The direct effect of connectivity on turning speed after accounting for both mediators was small (β = 0.133). The specific indirect effect through MSN was statistically significant (β = 0.312; 95% bootstrapped CI: [0.072, 0.605]; *p* = 0.008), while the indirect effect through VAF1 was non-significant (β = −0.067; 95% CI: [−0.273, 0.081]; *p* = 0.415). The contrast between the two indirect effects was significant (β = 0.379; 95% CI: [0.033, 0.832]; *p* = 0.028), confirming that MSN is the specific pathway through which cortical connectivity influences turning performance (Figure 6).

Furthermore, a statistically significant, moderate negative correlation was observed between gait speed and individual influence on the EEG–muscle synergy–gait pathway across all participants (R = −0.41, *p* = 0.03). This suggests that individuals with faster gait speeds were less reliant on the indirect cortical-to-motor pathway, whereas those with slower gait speeds relied more on this pathway. When examined within groups, healthy controls showed an insignificant positive association between gait speed and pathway influence (R = 0.51, *p* = 0.09). However, the PD group showed a strong, significant negative correlation (R = −0.82, *p* < 0.001), indicating greater reliance on the indirect pathway among those with reduced gait performance.

**Figure 7:**
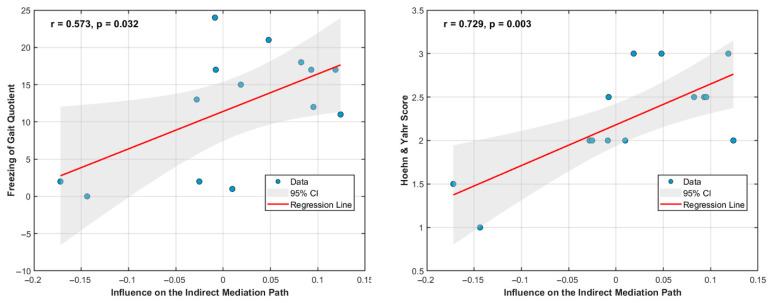
Correlational plots between the individual influence on the mediation pathway and the disease severity of PD participants. The more advanced the disease, the greater the individual’s reliance on the indirect cortical–synergy–gait pathway, suggesting a progressive shift toward compensatory top-down motor control in PD. Individuals with higher freezing-of-gait quotients rely more on the mediation pathway. Individuals with higher Hoehn and Yahr Scores also show higher reliance on the indirect mediation pathway. All other clinical scores showed weak, statistically insignificant associations with PD participants’ reliance on the mediation pathway (R < 0.3, *p* > 0.05).

Among PD participants, higher Freezing of Gait Questionnaire (FOGQ) scores were associated with greater influence on the indirect pathway (R = 0.57, *p* = 0.032). Similarly, higher Hoehn and Yahr (H&Y) stages were associated with greater reliance on the pathway (R = 0.73, *p* = 0.003), suggesting that individuals with more advanced PD increasingly depend on this compensatory control route (Figure 7).

## 4. Discussion

This study examined how cortical connectivity influences turning performance through muscle synergy organization in individuals with PD and healthy controls. We focused on a standardized 180° turning task, which is highly sensitive to PD-related gait deficits. Although broader locomotor contexts may enhance generalizability, restricting the analysis to a single, well-controlled task improved mechanistic precision in this pilot sample. By integrating cortical, neuromuscular, and biomechanical data, we identified a hierarchical control pathway linking cortical communication, muscle coordination, and turning performance.

Compared with healthy controls, individuals with PD demonstrated slower turns, shorter stride lengths, and reduced neuromuscular modularity, reflected in lower minimal synergy numbers and higher VAF1. These gait impairments reflect deficits in postural stability and bilateral coordination [3,4], while reduced muscle modularity indicates compensatory merging of synergies that limit adaptability during complex motor tasks such as turning [26,27,28]. The bilateral 8-muscle design employed here captures a more complete picture of turning biomechanics than the single-limb or symmetric configurations used in prior straight-walking synergy studies [31], where the distinct coordination demands of the inner and outer limbs are not preserved. Preserving the full bilateral set allowed synergy complexity to reflect the neural cost of coordinating both limbs simultaneously in PD. From the cortical connectivity, PD participants exhibited altered input strength to the frontal cortex during turning, reflecting a compensatory mechanism to offset the loss of automatic, subcortically mediated motor control [49,50]. Similar compensation patterns have been described in studies showing increased frontal activation and disruptions in β-band coherence during challenging gait tasks in PD [40,51,52]. Together, these results illustrate a progressive reorganization of motor control in PD, with weaker gait mechanics, stiffer muscle coordination, and impaired cortical network function compared with healthy controls.

Using a mediation framework to examine multimodal interactions among brain connectivity, muscle synergy complexity, and turning performance, we found that higher synergy complexity was associated with better turning performance, supporting the view that greater synergy complexity enables more flexible and adaptive movement [24,53,54]. Participants with higher MSN (mostly healthy individuals) showed superior turning performance compared to those with lower MSN. Reduced modularity and simplified muscle coordination, therefore, appear to underlie the turning gait difficulties observed in PD. The strong predictive power of the selected EEG connectivity for synergy complexity emphasizes the role of cortical communication in neuromuscular coordination. The strongest associations involved frontal and sensorimotor connections, consistent with their role in motor planning and execution [29,30]. Notably, many of the connections with high predictive coefficients were negative, indicating that increased connectivity in these regions was associated with reduced MSN and may reflect the compensatory processes in individuals with PD.

The Lasso-based mediation framework revealed that motor coordination, quantified by muscle synergy complexity, partially mediated the relationship between cortical connectivity and turning performance. The parallel mediation framework, which estimated both MSN and VAF1 pathways simultaneously, demonstrated that this mediating role was specific to MSN rather than synergy complexity broadly defined. While the PD group showed a trend toward higher VAF1, this metric appears insufficiently sensitive to detect the turning-specific coordination differences captured by MSN. MSN, as an explicit count of discrete functional modules, more directly reflects the neuromuscular complexity relevant to a biomechanically demanding bilateral task such as 180° turning. MSN functions effectively as a continuous mediator in bootstrapped regression-based frameworks, consistent with its established use in the neuromuscular literature [26,33], and the convergent non-significance of the VAF1 pathway suggests that the result is not an artifact of MSN’s measurement scale.

Connectivity strength across participants was associated with synergy complexity, which in turn predicted turning speed. The direct effect of cortical connectivity on turning speed, after accounting for synergy complexity, remained minimal, suggesting that a substantial portion of the brain’s impact on turning speed is mediated through neuromuscular coordination. While the regional connectivity analysis provides network-level characterization, the channel-level LASSO analysis identified specific directed connections that individually predicted synergy complexity. Prior work has established that reduced corticospinal coherence and disrupted frontal–sensorimotor oscillations are associated with slower gait and reduced step length in PD [15,51,52], yet these studies could not determine whether the brain acts on movement directly or through intermediate neuromuscular reorganization. Our findings extend this theory by identifying synergy complexity as a candidate intermediary mechanism through which cortical disruptions may contribute to impaired gait in PD. Thus, in PD, slower turning may result not only from changes in cortical dynamics but also from simplified downstream muscle coordination. Increased frontal input seems to decrease synergy complexity, reducing adaptive motor responses during turns.

Although PD participants were assessed on medication, the cortical–synergy–gait associations were consistent in direction and magnitude across all multimodal measures, which would be unlikely if the pattern were purely a pharmacological artifact. Studies have shown that levodopa modulates frontal connectivity and beta-band dynamics [55,56], and partially normalizes turning metrics in PD [57], meaning it could influence both the cortical predictor and the synergy mediator; ON/OFF crossover designs are needed to isolate this. Nonetheless, this pathway could be a therapeutic target, with interventions like motor rehabilitation or brain stimulation potentially improving turning in PD.

Building on this mediation framework, we further examined individual reliance on the indirect cortical–motor pathway (via EEG connectivity, muscle synergy, and turning speed). This pathway appeared to function as a compensatory mechanism predominantly used by individuals with PD. Participants with slower gait speeds and higher FOGQ and H&Y scores showed greater dependence on the cortical-synergy pathway than those with faster gait and lower disease severity. This pattern likely reflects a shift away from automatic gait control to a more consciously regulated strategy. In healthy individuals, gait is typically automatic and subcortically mediated, requiring minimal cortical involvement [40]. However, PD-related basal ganglia dysfunction disrupts this automaticity, prompting alterations in frontal cortical regions to sustain movement [52]. Previous studies similarly proposed that heightened frontal activation during gait in PD reflects a compensatory control, especially during complex or unstable walking conditions [52,58]. While this compensatory mechanism helps preserve mobility for a while, it may also reduce neuromuscular efficiency and flexibility, as evidenced by lower synergy complexity and impaired turning performance. Therefore, this research emphasizes the role of synergy complexity as a central element of motor control and offers a framework for understanding compensatory neural behavior in PD, which manifests as reduced movement adaptability.

## 5. Limitations and Future Study

Several methodological strengths support confidence in these findings, though important limitations should also be considered. The multimodal approach captured cortical activity via EEG, neuromuscular signals through EMG, and movement dynamics with motion capture, offering a comprehensive view beyond single-modality studies. Bootstrapped mediation analysis with 5000 resamples yielded robust results despite the small sample size typical of intensive EEG–EMG research. The bilateral 8-muscle EMG setup reflected the asymmetrical biomechanics of turning, not just averaging across limbs as in straight-walking studies. Limiting participants to individuals with PD who experience freezing episodes created a more homogeneous group, reducing variability and improving insights into underlying mechanisms.

Despite these strengths, several limitations should be considered. First, the sample size was modest, which is common in multimodal EEG–EMG–kinematic research where high data dimensionality and recording demands constrain recruitment. The observed effects were medium to large in magnitude, and the bootstrapped mediation framework provides robust inference under small samples; nevertheless, statistical power remains limited, and these findings should be treated as preliminary mechanistic evidence. Future studies with a larger sample size per group are recommended to confirm and extend these results.

Second, the PD sample included only individuals who experience freezing of gait. This creates a certain level of within-group homogeneity, which reduces a major source of gait variability in PD and strengthens the internal consistency of the results. However, generalizability to PD populations without freezing cannot be assumed, and future research should explore whether this cortical–synergy–gait pathway functions similarly across different PD subtypes. Specifically, future studies should involve non-freezing PD, tremor-dominant, and akinetic-rigid subtypes to determine if the cortical–synergy pathway applies across the full spectrum of PD motor phenotypes.

Third, All PD participants were assessed while in their typical ON-medication state, reflecting real-world walking conditions and allowing meaningful interpretation of motor performance. Levodopa doses varied widely. Further research is needed to isolate medication effects and ensure that the consistent relationships observed across cortical, muscular, and gait measures do not solely reflect medication. Future studies with a crossover ON/OFF design could directly evaluate dopaminergic effects on the cortical–synergy–gait pathway and distinguish between disease-related and medication-related influences on connectivity and synergy.

Fourth, only a standardized right-turn condition was analyzed. While this study controlled for directional variability and improved mechanistic precision, it limits generalizability to left turns and other locomotor contexts. Future studies should compare mediation strength across turn directions and gait tasks to test the specificity of these findings.

Finally, our cross-sectional design precludes causal inference. Longitudinal and interventional designs are needed to establish the directionality of influence across the cortical–neuromuscular–gait hierarchy, and to determine whether enhancing synergy complexity through rehabilitation translates to improved turning in PD.

## 6. Conclusions

This study demonstrates the hierarchical relationships among brain connectivity, muscle-synergy complexity, and turning performance in healthy and PD individuals. While previous research has examined brain-gait or muscle-gait relationships separately, primarily during straight walking, this study introduces a mediation framework that explicitly models how cortical disruption contributes to turning impairments—a fall-critical task that has been largely overlooked in neuromuscular studies. Our findings suggest that synergy complexity mediates the translation of cortical network disruptions into motor deficits. By integrating neurophysiological, muscular, and biomechanical data, we reveal a compensatory motor control pathway in PD, marked by an overreliance on cortical dynamics. As the disease progresses, this shift toward top-down control appears to undermine neuromuscular coordination, ultimately contributing to impaired gait. While preliminary, this work underscores the value of multimodal approaches for uncovering the mechanisms of motor dysfunction in PD. These insights highlight a novel therapeutic target: restoring effective brain–muscle communication by enhancing synergy complexity or modulating dysfunctional cortical connectivity. Such interventions may improve gait automaticity and provide a more efficient, flexible motor control strategy in individuals with PD. Importantly, given the cross-sectional design, our findings support a mechanistic hypothesis rather than causal inference, and replication in larger longitudinal samples is needed to establish directionality.

## Figures and Tables

**Table 1:** Clinical baseline characteristics of PD and healthy participant groups.

Characteristics	PD (*n* = 12)	Healthy (*n* = 12)
Age (years)	66.75 (6.90)	70.67 (5.53)
Height (cm)	165.0 (7.24)	165.0 (5.14)
Weight (kg)	64.42 (10.89)	64.19 (12.36)
Male/Female	7/5	8/4
Mini-Mental State Examination (MMSE)	26.85 (3.37)	27.71 (2.23)
Montreal Cognitive Assessment (MoCA)	22.71 (4.93)	27.29 (1.07)
Unified Parkinson’s Disease Rating Scale (UPDRS I, II, III)	55.42 (22.44)	N/A
Hoehn & Yahr (H&Y) Staging	2.39 (0.68)	N/A
Disease duration (years)	3.29 (2.75)	0
Freezing Of Gait Quotient (FOGQ)	15.79 (5.56)	0

**Table 2:** Group differences in gait, muscle synergy, and cortical connectivity measures.

Measure	Healthy (*n* = 12)		PD (*n* = 12)		Cohen’s d	*p*-Value
	*Mean*	*SD*	*Mean*	*SD*		
Gait Performance						
Turning Speed (m/s)	0.648	0.144	0.506	0.149	0.97	0.022 *
Stride Length (m)	0.767	0.169	0.597	0.213	0.89	0.035 *
Synergy Complexity						
First Variance Accounted For (VAF1)	0.711	0.046	0.755	0.062	−0.76	0.057
Minimal Synergy Number (MSN)	4.167	0.389	3.429	0.646	1.38	0.002 **
Cortical Connectivity						
Global Network Strength	3.494	0.961	3.000	1.342	0.423	0.214
Frontal → Frontal Strength	0.034	0.074	0.029	0.045	−1.031	0.017 *
Frontal → Central Strength	0.215	0.200	0.119	0.069	0.151	0.699
Frontal → Posterior Strength	0.148	0.181	0.078	0.093	−0.389	0.336
Central → Frontal Strength	0.022	0.085	0.028	0.081	−1.046	0.017 *
Central → Central Strength	—	—	—	—	—	—
Central → Posterior Strength	—	—	—	—	—	—
Posterior → Frontal Strength	0.222	0.148	0.091	0.074	0.884	0.033 *
Posterior → Central Strength	0.242	0.225	0.104	0.092	0.176	0.657
Posterior → Posterior Strength	0.117	0.087	0.063	0.042	0.566	0.157

* *p* < 0.05; ** *p* < 0.01. Dashes (—) indicate regional connectivity values of zero across all participants, reflecting the absence of edges surviving the consensus mask in that direction. Cohen’s d is reported as a signed effect size; negative values indicate a higher mean in the PD group.

## Data Availability

The datasets used and/or analyzed during the current study are available from the corresponding author on request.

## Abbreviations

PD	Parkinson’s Disease
EEG	Electroencephalography
EMG	Electromyography
MSN	Minimal Synergy Number (the smallest number of muscle synergies required to explain at least 90% of the total variance)
VAF1	Variance Accounted For using only one synergy
ICA	Independent Component Analysis
ASR	Artifact Subspace Reconstruction
EEGLAB	MATLAB toolbox for processing EEG data
NNMF	Non-Negative Matrix Factorization
PDC	Partial Directed Coherence
FOGQ	Freezing of Gait Quotient
H&Y	Hoehn & Yahr staging scores (PD disease severity scale)
UPDRS	Unified Parkinson’s Disease Rating Scale
MoCA	Montreal Cognitive Assessment
MMSE	Mini-Mental State Examination
LOO	Leave-One-Out (cross-validation method)
3D	Three-Dimensional
SENIAM	Surface Electromyography for the Non-Invasive Assessment of Muscles
R^2^	Coefficient of Determination (statistical measure of model fit)
CI	Confidence Interval
